# Higher expression of deoxyuridine triphosphatase (dUTPase) may predict the metastasis potential of colorectal cancer

**DOI:** 10.1136/jcp.2008.060004

**Published:** 2008-12-03

**Authors:** A Kawahara, Y Akagi, S Hattori, T Mizobe, K Shirouzu, M Ono, T Yanagawa, M Kuwano, M Kage

**Affiliations:** 1 Department of Diagnostic Pathology, Kurume University Hospital, Kurume, Japan; 2 Department of Surgery, Kurume University School of Medicine, Kurume, Japan; 3 Biostatistics Center, Kurume University, Kurume, Japan; 4 Department of Pharmaceutical Oncology, Graduate School of Pharmaceutical Sciences, Kyushu University, Fukuoka, Japan; 5 Center for Innovative Cancer Therapy, Kurume University, Kurume, Japan

## Abstract

**Aims:**

5-Fluorouracil (5-FU) is one of the most widely used anticancer drugs; however, the activity of 5-FU is determined by the presence of several enzymes that limit its activation or degradation, and these include dihydropyrimidine dehydrogenase (DPD), orotate phosphoribosyl transferase (OPRT), thymidylate synthase (TS), thymidine kinase (TK), thymidine phosphorylase (TP) and deoxyuridine triphosphatase (dUTPase). The aim of this study was to compare the expression levels of these enzymes between the primary colorectal cancer of patients with and without distant metastases. Furthermore, there was a comparison of these expression levels between the primary tumour and the corresponding metastasis.

**Methods:**

Of 55 patients with colorectal cancer, 20 had no metastasis and the other 35 had distant metastasis. A strong expression was classified as positive, while weak to moderate or no expression was negative by immunohistochemistry.

**Results:**

Of the six 5-FU-related enzymes, the numbers of patients with expression of dUTPase (54% versus 15%; p = 0.005), TK (26% versus 0%; p = 0.019) and DPD (17% versus 45%; p = 0.033) were significantly different in those with primary tumours with metastasis compared with those with non-metastasis, respectively. The altered expression of OPRT (34.3%), TS (40.0%) and dUTPase (42.9%) was significantly greater from primary to metastasis among the 35 patients with metastasis. By contrast, the expression of OPRT, TS and dUTPase was decreased in 6, 5 and 7 patients, respectively, in metastatic sites.

**Conclusions:**

From this comparative study of the six 5-FU-related enzymes in colorectal cancer, the expression of dUTPase was most significantly different between primary tumours and their corresponding metastatic tumour. It is suggested that dUTPase may be a predictive biomarker for the metastatic potential of colorectal cancer.

5-Fluorouracil (5-FU) is one of the most widely used anticancer drugs for the treatment of cancers of the colon, breast, skin, head and neck, stomach and liver.^1^ ^2^ 5-FU-based chemotherapeutic regimens have also become standard treatment for digestive tract cancers. However, to exert an anticancer effect, 5-FU must be intracellularly activated to inhibit thymidylate synthase (TS) activity, or be incorporated into RNA and/or DNA.^3^ Cancer cell sensitivity to 5-FU is often influenced by the presence of enzymes affecting anabolism and/or catabolism, and these enzymes include dihydropyrimidine dehydrogenase (DPD), orotate phosphoribosyl transferase (OPRT), thymidine phosphorylase (TP) uridine phosphorylase (UP) and thymidine kinase (TK).^3^ ^4^ Salonga *et al*^5^ have previously reported that overexpression of DPD, in addition to TP and TS, appears to be an important indicator of poor prognosis in patients with advanced colorectal cancer treated with 5-FU-based chemotherapy. Overexpression of deoxyuridine triphosphatase nucleotide hydrolase (dUTPase) is also significantly associated with poor prognosis in colorectal cancer patients treated with 5-FU.^6^

Among 5-FU-related enzymes, DPD, a rate-limiting enzyme involved in the degradation of pyrimidine base and pyrimidine-based antimetabolites, is well known to play a critical role in tumour responses and the general toxicity of 5-FU.^7^ In the human DPD gene (DPYP), 39 mutations and polymorphisms have been identified,^8^ ^9^ and patients with DPD-deficient mutations have been shown to suffer severe toxicity when treated with 5-FU.^10^ ^11^ On the other hand, overexpression of DPD is associated with the rapid metabolism of 5-FU resulting in its deactivation and weak clinical response. Kobunai *et al*^12^ have reported that low sensitivity to 5-FU-based drugs is significantly correlated with a higher copy number and mRNA levels of the human DPD gene in 31 human tumour xenografts, suggesting that DPD is a potent predictive biomarker for 5-FU-based chemotherapy. Except for DPD, however, the role of other enzymes in clinical responses to 5-FU or other relevant pyrimidine-based antimetabolites has not been well established. Identification of any predictive marker enzyme in 5-FU-related metabolism that could be associated with malignant progression in cancer may further contribute to the development of novel anticancer antimetabolites as well as potent 5-FU-related anticancer drugs.

It is also very important to understand whether any 5-FU-related enzymes are closely associated with malignant characteristics, including metastasis and invasion of colorectal cancer, to develop novel and potent antimetabolic anticancer therapeutic drugs. Novel molecular pathological diagnoses can also be developed to optimise 5-FU- or other antimetabolite-based chemotherapy against advanced colorectal cancer by targeting 5-FU-related enzymes specifically upregulated or downregulated in metastatic tumours. We will discuss which 5-FU-related enzyme could predict the metastatic potential of colorectal cancer.

## METHODS

### Patients and tumour samples

We examined 55 patients with primary colorectal cancer whose tumours had been completely surgically removed in the Department of Surgery of Kurume University, between 1999 and 2004 (table 1). Among 55 patients, 20 patients were free of metastases or recurrences after surgery, and the remaining 35 patients had distant metastases, and these included 18 patients with lung metastases and 17 patients with liver metastases; patients with both lung and liver metastases are not included. The 35 patients were histologically diagnosed as having metastatic colorectal cancers. The age of the patients with colorectal cancer ranged from 17 to 83 years (mean 64 years); 30 were men and 25 were women. There were 24 patients with rectal cancer and 31 patients with colon cancers. The mean follow-up was 68 months, with a range of 36–84 months. Immunohistochemical analysis of 5-FU-related enzymes, such as DPD, OPRT, TS, TK, TP and dUTPase, was performed on 90 tumour tissue samples: 55 primary tumour tissue samples, and 35 metastatic tissue samples of colorectal cancer to distant organs.

**Table 1:** Clinicopathological data of colorectal cancers

Characteristic	Patients with no metastasis (n = 20)	Patients with metastasis (n = 35)	p Value*
Age, median (range) (years)	68 (42–78)	65 (17–83)	0.916
Sex			
Male	10	20	0.779
Female	10	15	
Location			
Rectum	11	13	0.262
Colon	9	22	
Differentiation			
Good	11	21	0.781
Poor	9	14	
Primary tumour stage (T)			
T2	1	3	1.00
T3–4	19	32	
Adjuvant 5-FU-based chemotherapy	7	14	0.779
Yes	7	14	0.779
No	13	21	

*Calculated by Wilcoxon rank-sum test for age and by Fisher’s exact test for the other factors.

5-FU, 5-fluorouracil.

### Immunohistochemistry

The deepest invasive tissue samples of primary colorectal cancer and of the corresponding metastatic cancer with non-cancerous tissue margin were selected for immunohistochemistry. The expression of 5-FU-related enzymes was determined by using antibodies supplied by Taiho Pharmaceutical (Saitama, Japan) using a 4 μm section of paraffin-embedded tissue. Endogenous peroxidase activity was inhibited by incubating the slides in 3% H_2_O_2_ for 5 min. Antigen retrieval was performed by microwaving for 30 min in citrate buffer (pH 6.0) for dUTPase only. Each slide was incubated overnight with the antibody at 4°C. For staining detection, the ChemMate Envision method (DakoCytomation, Glostrup, Denmark) was used with diaminobenzidine as the chromogen.

### Evaluation of immunohistochemistry

The expression levels of DPD, OPRT, TS, TK and TP were each classified into two categories as negative or positive. Negative expression was accepted when a weak to moderate or no expression in the entire cytoplasm in >10% of cancer cells was observed. Positive expression was stated when a strong expression of the entire cytoplasm in >10% of cancer cells was present. The extent of staining of dUTPase was classified based on the percentage of cells with strongly stained nuclei: ⩾50% indicated that a cancer was positive, and ⩽49% or no expression indicated that it was negative.

### Statistical analysis

The association of metastasis status with clinicopathological characteristics was tested by Fisher’s exact test or Wilcoxon rank-sum test depending on the type of data. The association of the expression of 5-FU-related enzymes with primary tumours was tested by Fisher’s exact test. A p value of <0.05 was regarded as significant. The association between expression in primary tumours and in the corresponding metastasis are summarised descriptively using 2×2 tables.

## RESULTS

### Expression of 5-FU-related enzymes in non-cancerous and cancerous tissues of colorectal cancers

**Figure 1:**
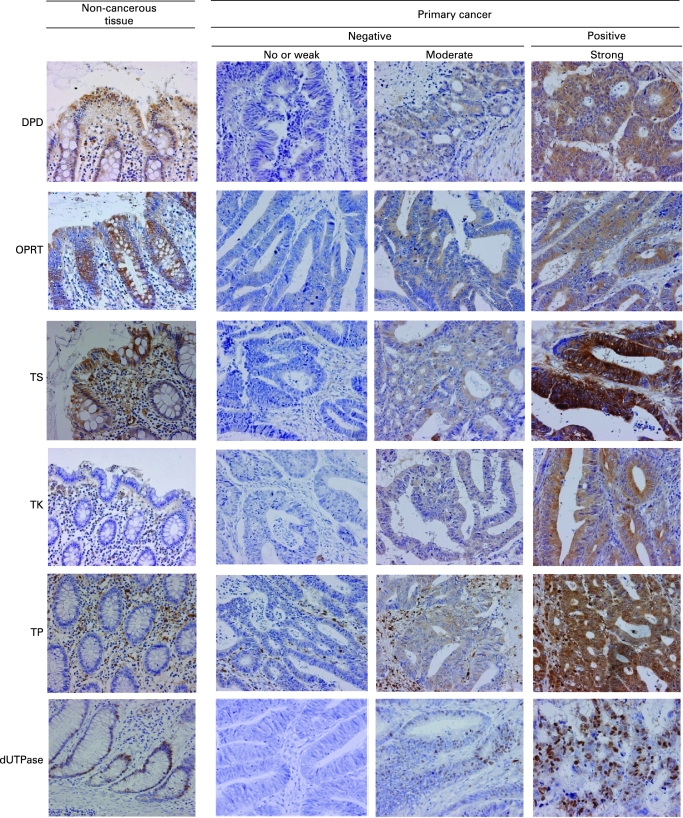
Expression of 5-fluorouracil-related enzymes in primary colorectal cancers and non-cancerous tissues. In primary tumours, expression levels were scored as negative (no or weak, or moderate, expression) and positive (strong expression). DPD, dihydropyrimidine dehydrogenase; dUTPase, deoxyuridine triphosphatase; OPRT, orotate phosphoribosyl transferase; TK, thymidine kinase; TP, thymidine phosphorylase; TS, thymidylate synthase.

We first examined whether six 5-FU-related enzymes were expressed in the non-cancerous epithelium of colorectal cancer by immunohistochemistry (fig 1). Of these six enzymes, DPD and TS were expressed in the surface and crypt epithelium. OPRT was expressed at various levels in the mucosa, and this enzyme was often highly expressed in the non-cancerous epithelium adjacent to cancer. TK and TP were not expressed in the non-cancerous epithelium, and TP was expressed in stromal components, including monocytes/macrophages. The expression of dUTPase was specifically observed in the nucleus of the lower part of the crypt epithelium and often in intracolorectal lymph nodes.

The expression of each of the six enzymes was classified as positive or negative in expression intensity assessed by immunohistochemistry, as shown in fig 1. Among 55 patients with colorectal cancers, DPD, OPRT, TS, TK, TP and dUTPase were positively expressed in 27% (15/55), 75% (41/55), 15% (8/55), 16% (9/55), 5% (3/55) and 40% (22/55) cases of primary tumours, respectively. The expression levels of OPRT and dUTPase in the primary tumours of patients with metastasis were higher relative to the expression of other enzymes (table 2). Furthermore, TK (p = 0.019) and dUTPase (p = 0.005) were expressed in higher levels in patients with metastases, whereas DPD (p = 0.033) was expressed in higher levels in patients without metastases.

**Table 2:** Comparison of 5-fluorouracil-related enzyme levels in primary tumours between patients with and without metastasis in colorectal cancer*

	Enzyme					
	DPD	OPRT	TS	TK	TP	dUTPase
Patients with metastasis (%)	17 (6/35)	83 (29/35)	20 (7/35)	26 (9/35)	9 (3/35)	54 (19/35)
Patients with no metastasis (%)	45 (9/20)	60 (12/20)	5 (1/20)	0 (0/20)	0 (0/20)	15 (3/20)
Fisher’s exact test, p value	0.033	0.106	0.234	0.019	0.293	0.005

*Fisher’s exact test was applied to 2×2 tables defined by marker status (+/−) and metastasis status to test the null hypothesis that the odds ratio was unity.

DPD, dihydropyrimidine dehydrogenase; dUTPase, deoxyuridine triphosphatase; OPRT, orotate phosphoribosyl transferase; TK, thymidine kinase; TP, thymidine phosphorylase; TS, thymidylate synthase.

### Alteration of expression of 5FU-related enzymes in primary tumours and corresponding metastases

We next compared the expression levels of 5-FU-related enzymes between primary tumours and those in corresponding metastatic tissues resected from the liver and lung, using immunohistochemistry. Non-cancerous liver and bile duct cells showed a weak to moderate expression of DPD and TS, but only a weak expression of OPRT, TK, TP and dUTPase. In contrast, the non-cancerous bronchial epithelium of the lung showed a weak to moderate expression of DPD, OPRT and TP, and almost no expression of TK and dUTPase. The basal cells of bronchial epithelium in the lung showed a strong expression of TS.

**Figure 2:**
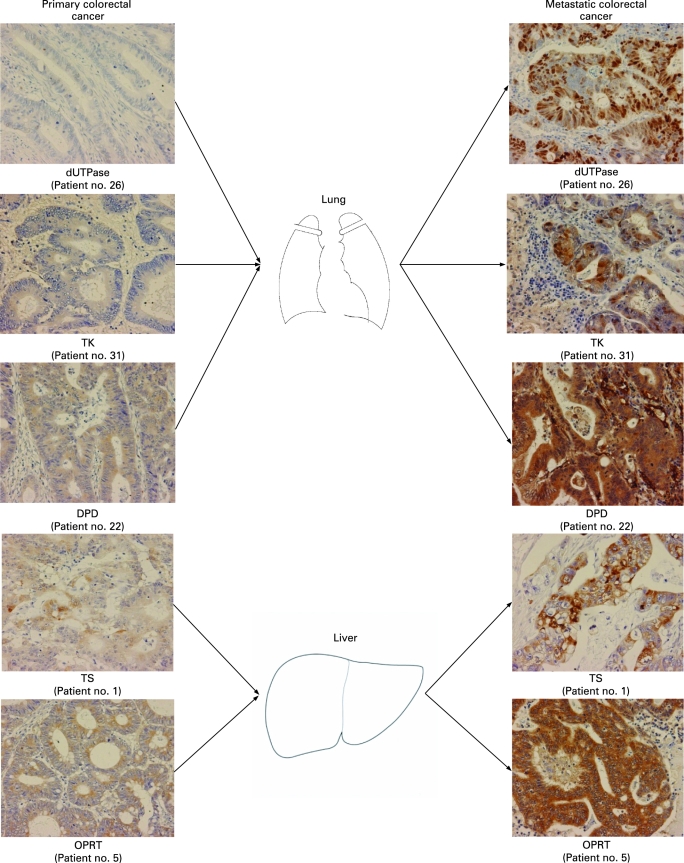
Comparison of the expression of 5-fluorouracil (5-FU)-related enzymes in primary colorectal cancers and colorectal metastatic cancers in five patients. In clinical specimens, the 5-FU-related enzymes showed a higher expression in metastases (lung in three patients and liver in two patients) than in their primary tumour counterparts. DPD, dihydropyrimidine dehydrogenase; dUTPase, deoxyuridine triphosphatase; OPRT, orotate phosphoribosyl transferase; TK, thymidine kinase; TP, thymidine phosphorylase; TS, thymidylate synthase.

Representative immunohistochemical data are shown in fig 2, and they indicate a relatively higher expression of six 5-FU-related enzymes in the metastases than in the primary tumours in five patients. Based on these immunohistochemical analyses of 35 clinical specimens, we examined whether the expression of these enzymes was increased or decreased in metastasis in comparison with their corresponding primary tumours. Metastatic colorectal cancers in the liver or lung showed a positive expression for DPD, OPRT, TS, TK, TP and dUTPase in 9% (3/35), 83% (29/35), 31% (11/35), 17% (6/35), 6% (2/35) and 57% (20/35) of cases, respectively (table 3). The expression of dUTPase and OPRT was found to be relatively higher and was scored more frequently as positive among the 5FU-related enzymes examined. Correlations between the expression of 5FU-related enzymes in the primary tumours and their corresponding metastatic tissues are summarised in 2×2 tables (table 3). In addition, the rates of positive expression in metastasis among patients with negatively expressed primary tumours and vice versa are also included. The altered relative ratios of enzyme expression in metastasis as compared with primary tumours are also presented. For each enzyme, expression levels in the metastasis were different relative to the primary tumours. In particular, for TS and dUTPase, 14 (40%) and 15 (42.9%) patients among 35 patients with metastasis experienced alternation of the enzyme expression levels in metastasis versus primary tumours. The relative ratios of enzyme expression in the metastasis were significantly altered in comparison to primary tumours for OPRT (6/6 = 100%) and dUTPase (8/16 = 50%).

**Table 3:** The expression of 5-fluorouracil-related enzymes between primary tumours and metastatic tumours

Enzyme	Primary site	Metastasis site			Altered expression*
		Negative	Positive	Total	
DPD	Negative	27	2	29	Increased: 2/29 = 6.9%
	Positive	5	1	6 (17%)	Decreased: 5/6 = 83.3%
	Total	32	3 (9%)	35	Altered: 7/35 = 20.0%
OPRT	Negative	0	6	6	Increased: 6/6 = 100%
	Positive	6	23	29 (83%)	Decreased: 6/29 = 20.7%
	Total	6	29 (83%)	35	Altered: 12/35 = 34.3%
TS	Negative	19	9	28	Increased: 9/28 = 32.1%
	Positive	5	2	7 (20%)	Decreased: 5/7 = 71.4%
	Total	24	11 (31%)	35	Altered: 14/35 = 40.0%
TK	Negative	23	3	26	Increased: 3/26 = 11.5%
	Positive	6	3	9 (26%)	Decreased: 6/9 = 66.7%
	Total	29	6 (17%)	35	Altered: 9/35 = 25.7%
TP	Negative	30	2	32	Increased: 2/32 = 6.3%
	Positive	3	0	3 (9%)	Decreased: 3/3 = 100%
	Total	33	2 (6%)	35	Altered: 5/35 = 14.3%
dUTPase	Negative	8	8	16	Increased: 8/16 = 50.0%
	Positive	7	12	19 (54%)	Decreased: 7/19 = 36.8%
	Total	15	20 (57%)	35	Altered: 15/35 = 42.9%

*Altered expression is indicated by determination of each enzymes expression in the primary tumour versus to the metastatic tumour. The rates of cases with positive or negative expression in the metastasis among cases with negative or positive expression in the primary tumour are indicated as increased or decreased. The rates of altered expression in the metastasis relative to that in the primary tumour are given in the row labelled as altered.

DPD, dihydropyrimidine dehydrogenase; dUTPase, deoxyuridine triphosphatase; OPRT, orotate phosphoribosyl transferase; TK, thymidine kinase; TP, thymidine phosphorylase; TS, thymidylate synthase.

## DISCUSSION

In our immunohistochemical analysis of six 5-FU-related enzymes (DPD, OPRT, TS, TK, TP and dUTPase), we compared the expression levels between the primary colorectal cancer and the metastasis in the lung or liver. The positive expression rate of dUTPase was higher in primary tumours with metastasis than in those without metastasis. We also observed a significant difference in the positive expression of DPD, TK and dUTPase between patients with and without metastasis. For each enzyme, the expression levels were different from those in the primary tumour and metastasis. The expression of TS and dUTPase in the metastasis was altered relative to that in the primary tumour in about 40% of patients. In particular, the expression of dUTPase in the primary tumour was most significantly increased in patients with metastatic colorectal cancers. dUTPase mediates a protective role against eventual toxicity occurring during DNA replication by reduction of the dUTP pool. Fleishman *et al*^13^ reported higher dUTPase expression in colorectal adenocarcinomas with lymph node metastasis compared with those without metastasis. A relevant study by Ladner *et al*^6^ also demonstrated that a low nuclear expression of nuclear dUTPase was associated with a better prognosis of patients after resection of colorectal cancer. The expression of dUTPase thus appears to be closely associated with the response to chemotherapy,^6^ an also with the metastatic potential of colorectal cancer, as revealed in this study. Therefore, evaluation of the expression of dUTPase in advanced metastatic cancer may be useful for selecting optimised therapies for patients with colorectal cancer. Furthermore, no therapeutic drug targeting dUTPase is used for cancer patients. Further development of dUTPase-targeting drugs might be useful for colorectal cancer with metastasis.

The expression of OPRT is known to be closely correlated with the therapeutic efficacy of a 5-FU-based drug in gastric cancer^14^ and colorectal cancer.^15^ OPRT was positive at the metastatic site from the primary lesion in which OPRT was negative in six patients (table 3); however, the expression of OPRT was relatively higher in primary tumours in patients with (83%) and without (60%) metastasis (table 2). Although OPRT is a key enzyme that might be responsible for the therapeutic efficacy of 5-FU-based chemotherapy, it seems less likely that OPRT predicts the metastatic potential of colorectal cancer.

**Take-home messages**

- The present study shows, for what is believed to be the first time, that expression of deoxyuridine triphosphatase (dTUPase) was augmented in metastasis as compared with the primary tumour in colon.
- Primary colorectal cancer showed a significantly higher expression of dUTPase in patients with metastasis when compared with dUTPase expression in patients without metastasis.
- The expression of dUTPase may thus predict the metastatic potential of colorectal cancer.

In conclusion, of 5-FU-related enzymes, primary tumours showed a significantly higher expression of dUTPase in patients with metastasis when compared with dUTPase expression in patients without metastasis. The expression of dUTPase was also increased in metastatic sites of eight of 16 patients with negative expression in their primary sites. The expression of dUTPase may thus predict the metastatic potential of colorectal cancer.
